# Malignant Mesothelioma of the Tunica Vaginalis: About a Rare Clinical Case

**DOI:** 10.7759/cureus.69897

**Published:** 2024-09-22

**Authors:** João Guerra, Joao M Pina, Vanessa Andrade, João Cassis, Luís Campos Pinheiro

**Affiliations:** 1 Urology, Centro Hospitalar Universitário de Lisboa Central, Lisbon, PRT; 2 Pathology, Hospital da Luz, Lisbon, PRT

**Keywords:** hydrocele, malignant mesothelioma, rare cancers, scrotal mass, tunica vaginalis

## Abstract

Malignant mesothelioma (MM) of the tunica vaginalis is an exceedingly rare neoplasm, with fewer than 300 cases reported in the medical literature. Due to its rarity, epidemiology, and risk factors are still unclear, and it is unknown whether asbestos or chronic inflammatory conditions play a role in etiology.

This case study presents a 70-year-old male patient with MM of the tunica vaginalis, detailing the diagnostic challenges, treatment procedures, and eventual progression to palliative care. The study underscores the importance of accurate diagnosis and the aggressive nature of the disease despite treatment efforts.

## Introduction

Malignant mesothelioma (MM) can develop from the coelomic epithelium at the pleura, peritoneum, pericardium, and tunica vaginalis testis. The latter is a rare and aggressive tumor, constituting only 0.3-5% of all MM cases [1-3]. It can occur at any age; however, the majority of patients are between 55 and 75 years of age [4,5]. This disease is associated with difficulty in diagnosis, high malignancy, easy recurrence, poor prognosis, and unclear epidemiological and risk factors [6,7]. This study aims to contribute to the limited body of knowledge on MM of the tunica vaginalis by presenting a clinical case and discussing the diagnostic process, treatment options, and prognosis.

## Case presentation

We describe the case of a 70-year-old man who, in 2020, presented with a painless increase in scrotal volume on the right side. The swelling had been progressively increasing over the course of several months. There was no associated erythema, trauma, or fever. The patient denied any significant urinary symptoms such as dysuria, hematuria, or changes in urinary frequency. He reported no history of similar symptoms on the contralateral side and the swelling remained unilateral, non-diffuse. The patient also noted no weight loss, anorexia, or systemic symptoms. His past medical history was notable for hypertension, managed with medication, but no prior urological surgeries or known history of malignancy and no exposure to asbestos. On physical examination, the scrotal swelling was firm, non-tender, and did transilluminate. A scrotal ultrasound was performed, compatible with a large right hydrocele.

In November 2020, the patient underwent surgical intervention for what was initially presumed to be a hydrocele. During the operation, multiple irregular, nodular vegetations were unexpectedly identified on the inner surface of the tunica vaginalis. These growths raised concern for a more sinister pathology. The abnormal tissue was meticulously excised and sent for histopathological examination.

Subsequent analysis of the excised tissue revealed the presence of a malignant neoplasm. Histologically, the tumor displayed a distinctive morphological pattern, characterized by diffuse atypical mesothelial cells forming papillary structures. Immunohistochemical staining was performed, which demonstrated strong positivity for markers consistent with mesothelioma, including calretinin and WT1, confirming the diagnosis of MM of the tunica vaginalis.

**Figure 1:**
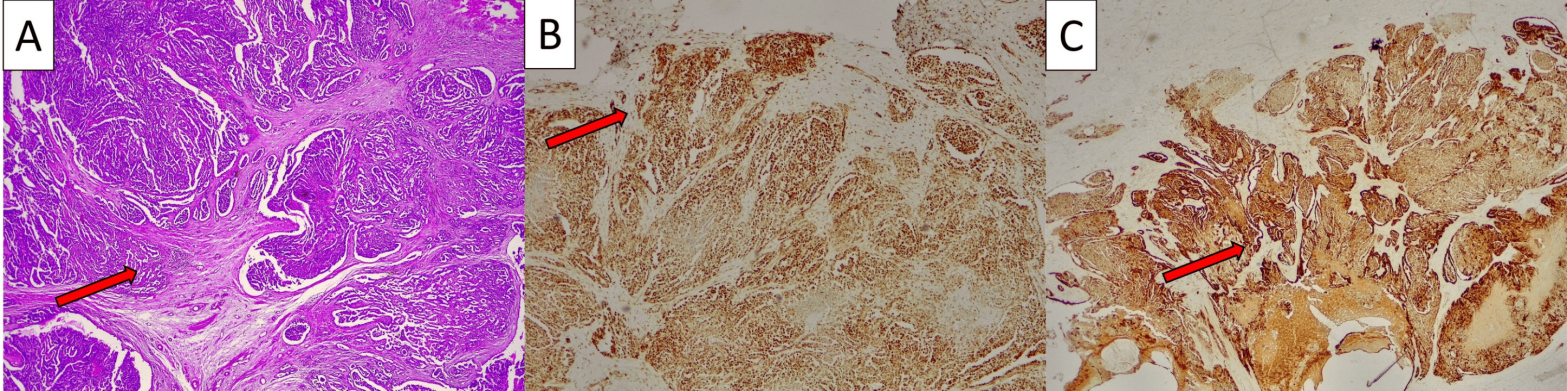
Histopathological examination of malignant mesothelioma A) H&E 25×, invasive malignant neoplasm with a micropapillary pattern, originating from the tunica vaginalis, invading the peritesticular soft tissues; B) WT1 25×, tumor cells are positive for WT1; C) Calretinin 25×, tumor cells are positive for calretinin, confirming the mesothelial nature of the neoplasm.

CT scan was performed without evidence of secondary deposits and PET-FDG describes increased metabolism in the right testicular tunica albuginea, compatible with already documented mesothelioma, and increased metabolism in inguinal ganglia, bilaterally, probably of a reactive nature. He was proposed for radical orchidectomy, which he underwent in December 2020. The histology of the surgical specimen confirms MM of the tunica vaginalis, diffuse, with a tubulo-papillary epithelial pattern, with focal invasion in the depth of the subcutaneous tissues of the scrotum, without invading the testicular parenchyma or other structures (Figure 1).

**Figure 2:**
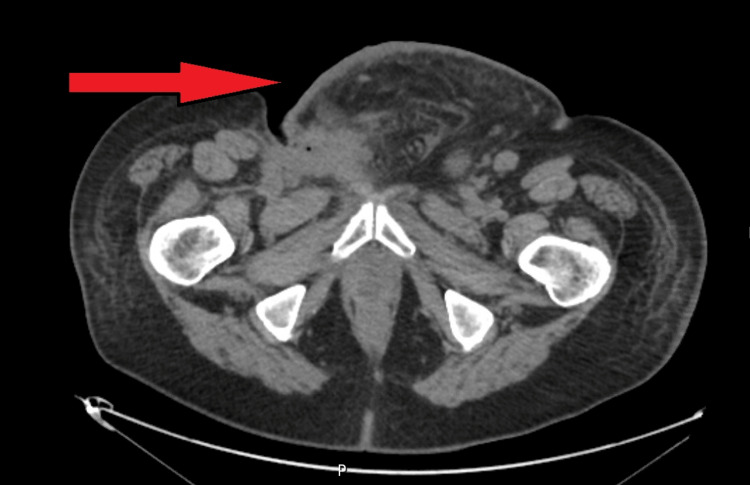
Abdominal CT image showing the tumoral pubic mass

In April 2021, suspicious right inguinal adenomegaly was found, and surgical excision of the same, after marking with a harpoon guided by ultrasound. It was a lymph node metastasis of MM of the testicular tunica vaginalis. Then he underwent block excision of a ganglionic mass in the right iliac fossa extending to the root of the ipsilateral thigh: neoplasia in the dermis and subcutaneous cellular tissue consistent with MM. Subsequently, signs of disease progression were evident in the October 2021 CT scan of lung and lymph node metastasis and the presence of an inflammatory mass in the inguinal and pubic region extending to the penis (Figure 2).

Biopsy performed by dermatology with evidence of infiltration of the dermis and hypodermis by MM.

Progressive clinical worsening, the clinical case re-discussed, and the decision to provide palliative care and comfort measures, with the patient dying in the following month.

## Discussion

MM of the tunica vaginalis is a rare entity that poses significant diagnostic and therapeutic challenges. The rarity of this condition has resulted in a limited number of case reports and series, with less than 300 cases documented worldwide, with the first case described by Barbera and Rubino in 1957 [8]. This scarcity of data hampers the understanding of its epidemiology and risk factors, particularly the role of asbestos exposure, which is well-established in pleural and peritoneal mesothelioma but remains uncertain in tunica vaginalis mesothelioma [4, 7]. Some suspected risk factors include trauma, herniorrhaphy, long-term hydrocele or spermatocele, long-term epididymitis, orchitis, or other inguinal inflammation [9,10]. It was suggested that it was linked to the production of interleukin-6, but the mechanism is not well established [11].

The clinical presentation of MM of the tunica vaginalis is often nonspecific, commonly mistaken for benign conditions such as hydrocele or other inguino-scrotal diseases. This lack of distinctive clinical features and tumor markers makes preoperative diagnosis exceedingly difficult. Imaging techniques like ultrasound and CT scans can provide suggestive findings but are not definitive [12]. Consequently, the diagnosis is frequently made postoperatively. Immunohistochemistry plays a critical role in distinguishing MM from other conditions and there are two key markers: WT1 and calretinin. WT1 is a transcription factor and calretinin is a calcium-binding protein both highly expressed in mesothelial cells. Their positivity strongly suggests mesothelial origin. Other mesothelial markers, such as D2-40 and CK5/6, provide additional diagnostic support, while carcinoembryonic antigen (CEA), Ber-EP4, and B72.3 are often negative in MM, but positive in metastatic adenocarcinomas, which further aids in the differentiation.

Our case aligns with the literature in that the patient presented with nonspecific symptoms and underwent surgery for presumed hydrocele, only to be found with multiple nodular vegetations on the tunica vaginalis mirroring findings reported in other cases. The histopathological examination, with the combination of WT1 and calretinin positivity, confirmed the diagnosis of MM.

The management of MM of the tunica vaginalis typically involves radical surgery, which may include orchidectomy and hemiscrotectomy, as radical procedures are believed to improve outcomes. However, the prognosis remains poor, with a high rate of local recurrence and metastasis, as evidenced by our patient's rapid disease progression despite aggressive surgical intervention and adjuvant therapies.

Adjuvant treatments, including chemotherapy with agents like cisplatin and pemetrexed, have been applied with varying success. In some cases, chemotherapy has been shown to slow progression, but the overall impact on survival is unclear [13,14]. The aggressive nature of MM of the tunica vaginalis and its propensity for early metastasis necessitate further research into more effective therapeutic strategies.

The case presented herein underscores the imperative for heightened clinical vigilance and consideration of MM of the tunica vaginalis in the differential diagnosis of inguino-scrotal swellings. It also highlights the importance of intraoperative findings and histopathological examination in securing a diagnosis. The rapid progression of the disease in our patient, despite no initial evidence of metastasis, illustrates the aggressive behavior of this malignancy and the need for ongoing surveillance post-treatment.

## Conclusions

MM of the tunica vaginalis, though rare, should be considered in the differential diagnosis of inguino-scrotal swellings. The case underscores the critical role of intraoperative vigilance and histopathological examination in achieving an accurate diagnosis. Despite comprehensive treatment, the prognosis remains poor, emphasizing the need for further research into effective therapeutic strategies.
